# Anemia Treatment and Its Clinical Implications in Patients Receiving Hemodialysis Hyporesponsive to Erythropoietin-Stimulating Agents

**DOI:** 10.1016/j.xkme.2026.101269

**Published:** 2026-01-22

**Authors:** James B. Wetmore, Julie Rouette, Anna Richards, Haifeng Guo, Gema Requena, Suying Li, George Mu, Liyuan Ma, David T. Gilbertson, Sally Wetten, Jiannong Liu

**Affiliations:** 1 Chronic Disease Research Group, Hennepin Healthcare Research Institute, Minneapolis, MN; 2 Division of Nephrology, Hennepin Healthcare, Minneapolis, MN; 3 GSK, Montreal, QC, Canada; 4 GSK, London, UK; 5 GSK, Collegeville, PA

**Keywords:** Anemia, erythropoietin-stimulating agents, health care utilization, hemodialysis, red blood cell transfusion, USRDS

## Abstract

**Rationale & Objective:**

Anemia, highly prevalent among patients receiving maintenance dialysis, may be associated with adverse outcomes. This study describes the patient characteristics, anemia treatment patterns, clinical event rates, and health care resource utilization of patients receiving hemodialysis (HD) in the United States and in a subpopulation of patients hyporesponsive to erythropoietin-stimulating agents (ESAs).

**Study Design:**

Using data from the US Renal Data System, a retrospective, population-based descriptive cohort study of adults with Medicare Parts A and B coverage receiving maintenance HD was conducted.

**Setting & Participants:**

ESA hyporesponsiveness was defined as ESA Resistance Index ≥2 (ESA units/kg/wk/g hemoglobin/L) or ESA dose ≥450 U/kg/wk (within 8 weeks of index (January 1, 2018).

**Outcomes:**

Anemia treatments and clinical events were assessed using medical claims during follow-up, until death, loss of Medicare, kidney transplantation, or December 31, 2019.

**Analytical Approach:**

Outcome rates were calculated as number of events divided by follow-up time with 95% confidence intervals based on Poisson distribution.

**Results:**

Of the overall prevalent HD population (N = 209,408), 20,223 were ESA hyporesponders. Relative to the overall prevalent HD population, the ESA hyporesponder subpopulation had a higher rate of red blood cell transfusion events (91.1 [95% CI, 90.1-92.2] vs 32.4 [95% CI, 32.2-32.6] per 100 person-years [PY]), thromboembolic events (80.7 [95% CI, 79.3-82.2] vs 71.0 [95% CI, 70.6-71.4] per 100 PY), major adverse cardiovascular events (30.2 [95% CI, 29.6-30.9] vs 20.3 [95% CI, 20.2-20.5] per 100 PY), gastrointestinal erosions (61.8 [95% CI, 60.6-63.0] vs 40.2 [95% CI, 40.0-40.5] per 100 PY), and all-cause mortality (26.2 [95% CI, 25.6-26.8] vs 16.9 [95% CI, 16.8-17.1] per 100 PY). Hospitalizations occurred more frequently in the ESA hyporesponder subpopulation (2.45 vs 1.56 per PY) than in the overall prevalent HD population.

**Limitations:**

This study was not designed to determine causality.

**Conclusions:**

The ESA hyporesponder subpopulation had a high burden of red blood cell transfusion use, clinical events, and health care resource utilization.

## Plain-Language Summary

Most patients receiving dialysis have anemia, which requires treatment. This study analyzed information on patients receiving maintenance hemodialysis in the United States and a subgroup of these patients whose anemia responded poorly to erythropoietin-stimulating agents (ESAs). Relative to the overall population of patients, those whose anemia responded poorly to ESAs had more frequent treatment with red blood cell transfusion, more hospitalizations, higher cardiovascular and non-cardiovascular health complications, and higher rates of death. The findings highlight the importance of understanding outcomes in patients whose anemia responded poorly to ESAs and the need to treat these patients appropriately, which may help inform future resource planning for the health care system.

Anemia is highly prevalent among patients receiving maintenance hemodialysis (HD).^1^^,^^2^ Anemia is a substantial burden on patients’ lives and has a negative impact on health-related quality of life.^3^ For decades, the management of anemia has been limited to erythropoietin-stimulating agents (ESAs), intravenous (IV) iron, and, as a last resort, red blood cell (RBC) transfusions.^1^^,^^4^ Despite guidelines on the management of anemia, the specifics of anemia management often vary depending on dialysis modality, individual patient characteristics, and local clinical practice patterns.^5^^,^^6^

A particular challenge in anemia management is posed by patients who are poorly responsive to ESAs (“ESA hyporesponders”).^7^ The reported prevalence of ESA hyporesponders varies considerably, due in part to a lack of consensus and consistency in the definitions of ESA hyporeponsiveness.^8^^,^^9^ Hyporesponsiveness has been variously defined as the inability to achieve a target hemoglobin level below a threshold ESA dose,^10^ the need for more than 300 IU/kg epoetin (or, equivalently, 1.5 μg/kg darbepoetin) per week,^11^ or by a high ESA Resistance Index.^12^^,^^13^ An ESA Resistance Index ≥2 (measured as ESA units/kg/wk/g hemoglobin/L) or an IV erythropoietin equivalent dose of ≥450 U/kg/wk has often been used to define ESA hyporesponsiveness.^12^^,^^13^ According to 2 studies in the United States that used an ESA Resistance Index-based definition of ESA hyporesponsiveness, 11%-13% of patients receiving maintenance dialysis are hyporesponsive.^8^

ESA hyporesponsiveness is typically transient because common conditions that contribute to hyporesponsiveness may themselves be amenable to treatment^14^; for example, chronic inflammation, advanced hyperparathyroidism, inadequate dialysis, and iron deficiency are highly prevalent, but potentially treatable, causes of ESA hyporesponsiveness.^10^^,^^14^^,^^15^ A recent study of patients receiving dialysis using data from the US Renal Data System (USRDS) classified ESA hyporesponders based on the pattern of hyporesponsive months during the study period.^13^ Of over 400,000 patients, 40% were found to be hyporesponsive to ESAs; of these hyporesponders who met the inclusion criteria (over 43,000), nearly half (45%) were considered “intermittent” (<75% of the study period with hyporesponsive months), a quarter (24%) were “isolated” (≤2 consecutive hyporesponsive months with no additional hyporesponsive months during the study period), and 1 in 5 (21%) were “chronic” (≥75% of the study period with hyporesponsive months).^13^

Despite the prevalence of ESA hyporesponders in the United States, few studies have examined the patient characteristics, anemia treatment approaches, and occurrence of clinical events in these patients. One US study reported that individuals with ESA hyporesponsiveness had a higher comorbidity burden, higher mortality rate, higher rates of hospitalization for cardiovascular events, and higher health care costs relative to individuals who were normoresponsive.^13^ Evidence gaps remain on the patterns of anemia treatment and rates of both cardiovascular and non-cardiovascular clinical events and health care resource utilization (HCRU) in more recent cohorts of patients who are hyporesponsive to ESAs. In an era of evolving treatment options for anemia of kidney disease, there is a need to better understand the clinical implications of ESA hyporesponsiveness. This study aimed to describe patient characteristics, patterns of anemia treatment, cardiovascular and non-cardiovascular clinical event rates, and HCRU in the overall prevalent HD patient population in the United States, and in a subpopulation of these patients who were hyporesponsive to ESAs to better understand the implications of ESA hyporesponsiveness.

## Methods

### Data Sources

The 2017-2019 USRDS database was used for this study. The USRDS database includes Medicare claims data and US End-Stage Renal Disease Quality Reporting System data from the Centers for Medicare & Medicaid Services. Medicare claims (billing) are for services from institutional settings (eg, inpatient, outpatient, skilled nursing facility, home health, and hospice), physician services, and prescriptions of medications and durable medical equipment, and prescription medications for medical beneficiaries. These claims include diagnosis, procedure, and revenue center codes with their respective dates of service, as well as the specialty of the treating provider. The claims also contain Healthcare Common Procedure Coding System (HCPCS) or National Drug Code information for prescription drugs, which includes their dosages and days of drug administration or days supplied. US End-Stage Renal Disease Quality Reporting System data, which cover all US patients receiving maintenance dialysis, include detailed dialysis-related information such as dialysis initiation date, reason for receiving maintenance HD, comorbid conditions at dialysis initiation, dialysis modality and setting, select laboratory results, anemia treatments, and date and cause of death. Together, these data sources provide comprehensive information on patient characteristics, dialysis-related information, and outcomes of interest.

### Study Design

This was a retrospective, observational, descriptive cohort study of adult Medicare fee-for-service beneficiaries receiving maintenance HD.

### Inclusion and Exclusion Criteria

To be included, patients were aged ≥18 years and on HD on January 1, 2018 (index date) and covered by Medicare Parts A and B in the 6 months prior to and on the index date. Follow-up was from the index date to the date of death, loss of Medicare fee-for-service coverage, the day before kidney transplantation, or December 31, 2019, whichever came first. Exclusion criteria included individuals with a functioning kidney transplant before the index date or who had cancer within 1 year prior to or on the index date, or hospitalization for heart failure, myocardial infraction, or stroke within 4 weeks prior to index date.

### Study Population

From the overall prevalent HD population described above, a subgroup of patients was classified as hyporesponsive to ESAs. Hyporesponsiveness was defined as having (1) an ESA Resistance Index ≥2 units/kg/wk/g hemoglobin/L or (2) an ESA dose ≥450 U/kg/wk in the 8 weeks prior to index date. This was based on the definition of ESA hyporesponsiveness used in previously conducted clinical trials.^12^^,^^13^^,^^16^ ESA doses were converted to IV epoetin alfa equivalence (where 1 unit of subcutaneous epoetin alfa ≈1.42 units of IV epoetin alfa; 1 μg of epoetin beta ≈208 units IV epoetin alfa; and 1 μg darbepoetin alfa ≈250 units of IV epoetin alfa).^12^^,^^13^^,^^16^

### Study Outcome Measures

Outcomes included rates of anemia treatments (use of ESAs, IV iron infusions, and RBC transfusions), rates of key clinical events of interest, and HCRU in the follow-up period, as described in detail below.

### Anemia Management

Management of anemia assessed during follow-up included the use of ESAs, IV iron infusions, and RBC transfusions. ESA use was identified by HCPCS codes from dialysis facility claims, which included those for epoetin alfa, epoetin beta, and darbepoetin alfa administration. ESA use was measured as days covered by ESA per week: 3 administrations of epoetin alfa was categorized as 7 days of coverage (3 times per week), 1 epoetin beta administration as 14 days of coverage (once every 2 weeks), and 1 darbepoetin administration as 7 days (once a week). The coverage was calculated for each patient as total days covered divided by the total days of follow-up, multiplied by 7. Similarly, IV iron use was defined by HCPCS codes from dialysis facility claims and measured as the number of administrations per week. RBC transfusions were identified from claims using revenue center codes, *International Classification of Diseases, Tenth Revision* procedure codes, and Current Procedural Terminology or HCPCS codes. Transfusions were reported as number per 100 person-years (PY). Multiple records of transfusions on the same day were counted as 1 transfusion event.

### Clinical Events

Clinical events of interest were drawn from those used in previously conducted clinical trials^12^^,^^17^ and included all-cause mortality and key cardiovascular and non-cardiovascular events. Cardiovascular events included major adverse cardiovascular events (MACE; defined as all-cause mortality, myocardial infarction, and stroke), MACE + heart failure, heart failure alone, and/or thromboembolic events (defined as vascular access thrombosis, deep vein thrombosis, and pulmonary embolism). Non-cardiovascular events included malignancies, serious infections, seizures, esophageal and gastric erosions, hepatic injury, retinal hemorrhage, and other ocular events.

Outcomes were derived from medical claims in the follow-up period using the *International Classification of Diseases, Tenth Revision* diagnosis, HCPCS, and/or Current Procedural Terminology codes. Myocardial infarction, stroke, heart failure, seizures, pulmonary embolism, and serious infections were ascertained from inpatient claims only. Deep vein thrombosis, malignancies, and esophageal and gastric erosions required 1 inpatient or 2 outpatient/Part B claims using specific diagnosis code algorithms. Retinal hemorrhage and other ocular events, as well as hepatic injury, required 1 claim of any type. First occurrence of the event during follow-up was captured for each outcome.

### HCRU

HCRU was identified in terms of care service setting and physician specialty. Care setting consisted of any hospitalization (inpatient admission), hospitalization requiring use of an intensive care unit (a subset of hospitalization), hospitalization requiring an intermediate care unit (“step-down” unit), hospital observation stay (typically, a brief stay not technically classified as an admission), stay in a skilled nursing facility, emergency department encounter not leading to a hospitalization, and outpatient encounter. Physician specialty was classified as nephrologist, cardiologist, endocrinologist, and/or primary care provider (internal medicine or family practice). HCRU was measured as number of encounters per PY of follow-up, expressed as rate.

### Statistical Analysis

Patient baseline characteristics were tabulated for the ESA hyporesponder subpopulation and the overall cohort. Descriptive statistics were used to summarize patient characteristics; mean and standard deviation (SD) or median and interquartile range (IQR) were reported for continuous variables, and percentages were reported for categorial variables. For clinical events, first clinical event rates were calculated as the number of events per 100 PY with associated 95% confidence interval (CI). Anemia treatment was calculated as weighted means of patient-level measures using follow-up as weight. For clinical events and anemia treatment, the 95% CIs were calculated based on the Poisson distribution.

## Results

### Patient Baseline Characteristics and Comorbid Conditions

**Figure 1:**
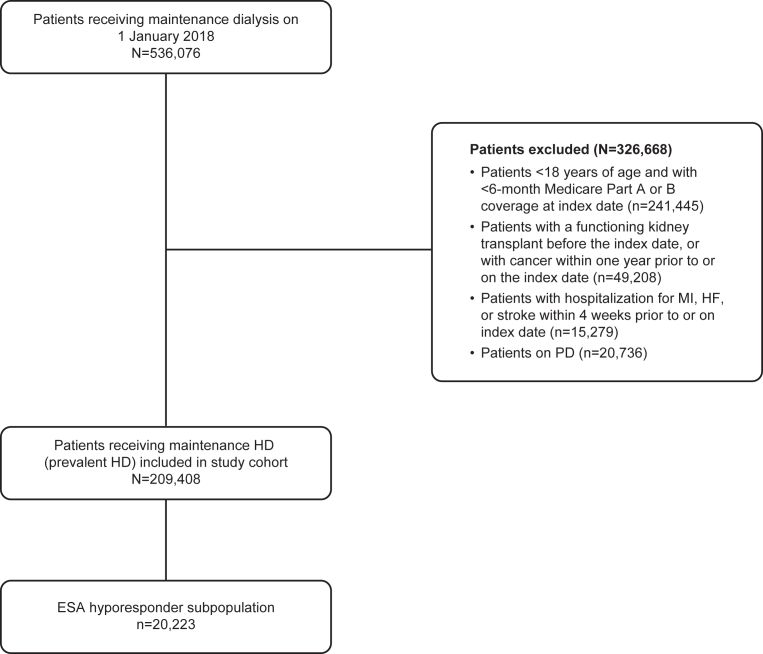
Study flow chart. Abbreviations: ESA, erythropoietin-stimulating agent; HD, hemodialysis; HF, heart failure; MI, myocardial infarction; PD, peritoneal dialysis.

In total, 209,408 patients were included in the overall prevalent HD population, of whom 20,223 (9.7%) were defined as ESA hyporesponders (Fig 1). Relative to the overall prevalent HD population, the subgroup who were hyporesponsive to ESA were more commonly female (53.2% vs 43.5%), non-Hispanic Black (41.4% vs 37.7%), dually enrolled in Medicare and Medicaid (54.0% vs 48.7%), and less likely to have diabetes as the reason for receiving maintenance HD (44.2% vs 49.3%). ESA hyporesponders had a longer dialysis duration than the overall prevalent HD population (4.5 vs 3.9 years). Most comorbid conditions were more common in the ESA hyporesponder subpopulation than in the overall prevalent HD population (Table 1).

**Table 1:** Patient Baseline Characteristics and Comorbid Conditions Among the Overall Prevalent HD Population and the ESA Hyporesponder Subpopulation

Baseline^a^ Characteristics	Overall Prevalent HD Population N = 209,408	ESA Hyporesponder Subpopulation n = 20,223
Age, y, mean (SD)	63.6 (13.8)	62.3 (14.8)
Sex		
Male, n (%)	118,310 (56.5%)	9,456 (46.8%)
Race/ethnicity, n (%)		
Non-Hispanic White	81,812 (39.1%)	7,119 (35.2%)
Non-Hispanic Black	78,930 (37.7%)	8,366 (41.4%)
Hispanic	33,810 (16.1%)	3,021 (14.9%)
Other	14,856 (7.1%)	1,717 (8.5%)
Residence area, n (%)		
Large metropolitan area	172,852 (82.5%)	17,143 (84.8%)
Non-large metropolitan area	36,556 (17.5%)	3,080 (15.2%)
Medicare/Medicaid dual enrollment, n (%)		
Yes	101,957 (48.7%)	10,929 (54.0%)
Reason for receiving maintenance HD, n (%)		
Diabetes	103,138 (49.3%)	8,937 (44.2%)
Hypertension	63,987 (30.6%)	6,373 (31.5%)
Glomerulonephritis	17,801 (8.5%)	2,234 (11.1%)
Other/unknown/missing	24,482 (11.7%)	2,679 (13.3%)
Dialysis duration, y, median (IQR)	3.9 (1.9-6.8)	4.5 (2.3-7.4)
Comorbid conditions, n (%)		
Diabetes	142,594 (68.1%)	13,153 (65.0%)
Atherosclerotic heart disease	72,191 (34.5%)	8,542 (42.2%)
Acute myocardial infarction	17,285 (8.3%)	2,605 (12.9%)
Heart failure	85,896 (41.0%)	10,172 (50.3%)
Dysrhythmia	46,463 (22.2%)	6,081 (30.1%)
Other cardiac diseases	55,097 (26.3%)	7,418 (36.7%)
Stroke or transient ischemic attack	30,674 (14.7%)	3,602 (17.8%)
Peripheral vascular disease	64,168 (30.6%)	7,726 (38.2%)
Pulmonary embolism	259 (0.1%)	49 (0.2%)
Deep vein thrombosis	4,218 (2.0%)	686 (3.4%)
Vascular access thrombosis	80,774 (38.6%)	8,451 (41.8%)
Chronic obstructive pulmonary disease	40,829 (19.5%)	5,226 (25.8%)
Liver disease	15,205 (7.3%)	2,132 (10.5%)
Gastrointestinal disease	9,476 (4.5%)	2,301 (11.4%)
Esophageal and gastric erosions	39,344 (18.8%)	6,231 (30.8%)
Seizures	5,891 (2.8%)	1,227 (6.1%)
Serious infections	21,416 (10.2%)	3,704 (18.3%)
Retinal hemorrhage	8,157 (3.9%%)	754 (3.7%)
Ocular events	46,356 (22.1%)	4,359 (21.6%)

Abbreviations: ESA, erythropoietin-stimulating agent; HD, hemodialysis; IQR, interquartile range; SD, standard deviation.

a Baseline period was 6 months prior to index date (January 1, 2018).

Relative to the overall prevalent HD population, the ESA hyporesponder subpopulation showed lower levels of hemoglobin (median 9.9 [IQR, 9.1-10.6] g/dL vs 10.8 [IQR, 10.2-11.4] g/dL), lower transferrin saturation (TSAT) (median 24.0 [IQR, 18.0-31.0] g/dL vs 30.0 [IQR, 23.0-38.0] g/dL), and a higher level of iron deficiency (TSAT <20% [IQR, 30.5% vs 13.0%]) at baseline (Table 2).

**Table 2:** Baseline Laboratory Values

Baseline^a^ Laboratory Values	Overall Prevalent HD Population N = 209,408	ESA Hyporesponder Subpopulation n = 20,223
Hemoglobin values^b^, g/dL, median (IQR)	10.8 (10.2-11.4)	9.9 (9.1-10.6)
Hemoglobin values, g/dL, n (%)		
<8.0	2,286 (1.1%)	1,227 (6.1%)
8.0 to <9.5	17,915 (8.6%)	5,946 (29.4%)
9.5 to <11.0	98,352 (47.0%)	10,517 (52.0%)
≥11.0	85,809 (41.0%)	2,531 (12.5%)
Missing	5,046 (2.4%)	NR^c^
Ferritin values^b^, ng/mL, median (IQR)	845.0 (550.0-1,159.0)	735.0 (443.0-1,059.0)
Ferritin values, ng/mL, n (%)		
<600	57,034 (27.2%)	7,426 (36.7%)
600-1000	69,681 (33.3%)	6,658 (32.9%)
>1000	72,968 (34.8%)	5,608 (27.7%)
Missing	9,725 (4.6%)	531 (2.6%)
Transferrin saturation^b^, median (IQR)	30.0 (23.0-38.0)	24.0 (18.0-31.0)
Transferrin saturation groups, n (%)		
<20	27,216 (13.0%)	6,171 (30.5%)
20-40	130,936 (62.5%)	11,362 (56.2%)
>40	43,012 (20.5%)	2,248 (11.1%)
Missing	8,244 (3.9%)	442 (2.2%)

Abbreviations: ESA, erythropoietin-stimulating agent; HD, hemodialysis; IQR, interquartile range; NR, not reported.

a Baseline period was 6 months prior to index date (January 1, 2018).

b For patients with data available.

c <11 patients, as per database policy.

### Anemia Treatment in the Follow-up Period by Patient Demographic

Use of each assessed anemia treatment in the follow-up period was higher in the ESA hyporesponder subpopulation than in the overall prevalent HD population. Relative to the overall prevalent HD population, ESA use (shown as days covered per week) was nearly twice as high (5.24 [95% CI, 5.23-5.24] vs 2.64 [95% CI, 2.63-2.64]) and the RBC transfusion rate nearly 3 times as high (91.1 [95% CI, 90.1-92.2] vs 32.4 [95% CI, 32.2-32.6] per 100 PY) for the ESA hyporesponder subpopulation (Table 3). Relative to the overall prevalent HD population, rate of IV iron use (shown as administrations per week) was slightly higher among the ESA hyporesponders (0.57 [95% CI, 0.57-0.57] vs 0.48 [95% CI, 0.48-0.48] per 100 PY) (Table 3). The use of anemia treatment by race and ethnicity, sex, and age subgroups is shown in Table S1.

**Table 3:** Anemia Treatment in the Follow-up Period

Characteristic	Overall Prevalent HD Population N = 209,408	ESA Hyporesponder Subpopulation n = 20,223
	Anemia Treatment During Follow-up, Weighted Mean (95% CI)	
ESA use^a^, days covered per week	2.64 (2.63-2.64)	5.24 (5.23-5.24)
IV iron use, administrations per week	0.48 (0.48-0.48)	0.57 (0.57-0.57)
RBC transfusion rate, per 100 PY	32.4 (32.2-32.6)	91.1 (90.1-92.2)

Abbreviations: CI, confidence interval; ESA, erythropoietin-stimulating agent; HD, hemodialysis; IV, intravenous; PY, person-years; RBC, red blood cell.

a ESA use was measured as days covered by ESAs per week: 3 administrations of epoetin alfa were categorized as 7 days of coverage; 1 epoetin beta as 14 days of coverage; and 1 darbepoetin administration was categorized as 7 days.

### Cardiovascular and Non-cardiovascular Clinical Event Rates in Follow-up

**Figure 2:**
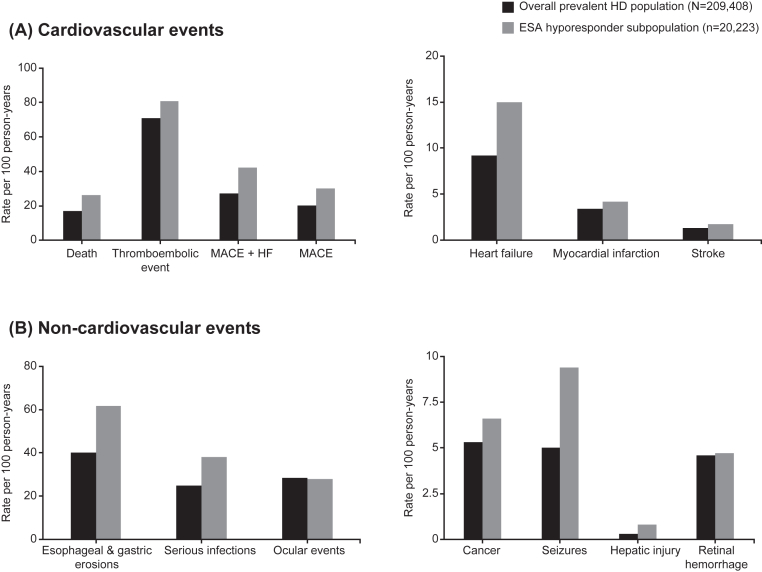
Rate of cardiovascular and non-cardiovascular events. (A) Rates of cardiovascular events per 100 PY (B) Rates of non-cardiovascular events per 100 PY. Abbreviations: ESA, erythropoietin-stimulating agent; HF, heart failure; MACE, major adverse cardiovascular event.

The ESA hyporesponder subpopulation generally showed higher unadjusted rates of cardiovascular events per 100 PY relative to the overall prevalent HD population for thromboembolic events (80.7 [95% CI, 79.3-82.2] vs 71.0 [95% CI, 70.6-71.4]); MACE (30.2 [95% CI, 29.6-30.9] vs 20.3 [95% CI, 20.2-20.5]) and MACE + heart failure (42.1 [95% CI, 41.3-42.9] vs 27.1 [95% CI, 26.9-27.3]) (Fig 2, Table S2). Similarly, relative to the overall prevalent HD population, higher unadjusted rates of non-cardiovascular events were observed for the ESA hyporesponder subpopulation for esophageal and gastric erosions (61.8 [95% CI, 60.6-63.0] vs 40.2 [95% CI, 40.0-40.5] per 100 PY) (Fig 2, Table S2). Unadjusted rates of all-cause mortality were higher for the ESA hyporesponder subpopulation (26.2 [95% CI, 25.6-26.8] per 100 PY vs 16.9 [95% CI, 16.8-17.1] per 100 PY), relative to the overall prevalent HD population (Fig 2, Table S2).

#### HCRU Rates During Follow-up Period

**Figure 3:**
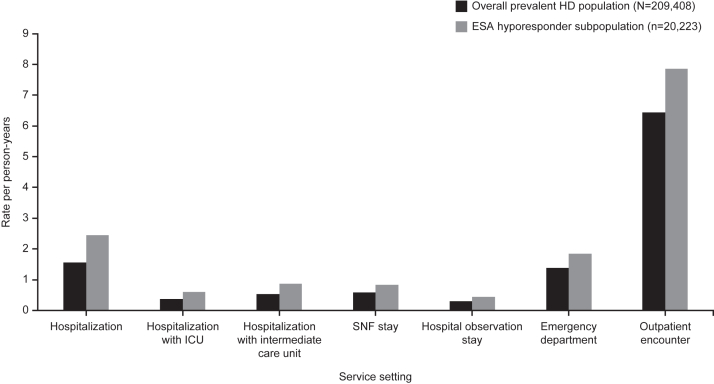
Rates of health care resource utilization in the ESA hyporesponder subpopulation per person-year. Abbreviations: ED, emergency department; ESA, erythropoietin-stimulating agent; HD, hemodialysis; ICU, intensive care unit; PY, person-years; SNF, skilled nursing facility.

Relative to the overall prevalent HD population, rates were higher in the subpopulation of patients with hyporesponsiveness for all-cause hospitalization (2.45 vs 1.56 per PY), hospitalization requiring use of the intensive care unit (0.61 vs 0.37 per PY), hospitalization requiring use of an intermediate care (“step-down”) unit (0.86 vs 0.54 per PY), observation stays (0.45 vs 0.30 per PY), emergency department encounters (1.85 vs 1.39 per PY), and total outpatient encounters (7.85 vs 6.44 per PY) (Fig 3, Table S3). Use of skilled nursing facilities upon discharge per PY were also higher in the ESA hyporesponder subpopulation (0.84 vs 0.59) than in the overall prevalent HD population. Patients in the ESA hyporesponder subpopulation visited physicians more often per PY than the overall prevalent HD population, most notably nephrologists (18.77 vs 15.55), primary care providers (14.40 vs 10.55), and cardiologists (5.15 vs 4.10) (Table S3).

## Discussion

ESA hyporesponsiveness is a major challenge for individuals receiving maintenance dialysis. Given the changing landscape of anemia treatment in the United States and the potential for new treatments,^18^^,^^19^ we sought to describe patient characteristics, anemia treatment patterns, key clinical outcomes, and utilization of health care resources in a large population of individuals receiving maintenance HD and in a subpopulation of those individuals who were hyporesponsive to ESAs. We found that the subpopulation of ESA hyporesponders differed in their demographic and clinical characteristics relative to the overall prevalent HD population, had a generally higher comorbidity burden, and, on average, had lower hemoglobin levels and iron saturation percentages at baseline, consistent with the findings of a previous report.^13^ The ESA hyporesponders also required a higher use of RBC transfusions than the overall prevalent HD population. Rates of all-cause mortality, most clinical outcomes, and HCRU were higher in the ESA hyporesponder subpopulation. These findings suggest that individuals with ESA hyporesponsiveness have a distinct clinical profile, different anemia treatment experiences, and may incur profound downstream clinical consequences.

Relative to the overall prevalent HD population, individuals with ESA hyporesponsiveness were more commonly women and of non-Hispanic Black race/ethnicity. Generally, women have lower hemoglobin levels than men and Black people lower levels than White people.^20^^,^^21^ Therefore, some commonly used definitions of ESA hyporesponsiveness may classify individuals differently by sex, race, and ethnicity. As such, these definitions, which do not explicitly consider these key demographic factors, may not be equally useful or informative when applied in the same manner to all patients. This is a potential avenue for future research.

With the exception of diabetes, the prevalence of most comorbid conditions was higher in the ESA hyporesponder subpopulation than in the overall prevalent HD population. This was the case not only for manifestations of cardiovascular disease such as heart failure, atherosclerotic heart disease, and peripheral vascular disease, but also for a variety of non-cardiovascular clinical events, including serious infections, chronic obstructive pulmonary disease, and gastrointestinal disease. The greater collective presence of these comorbid conditions, therefore, may be related to the presence of ESA hyporesponsiveness, either through direct mechanisms, such as inflammation,^10^ or through other mechanisms yet to be fully characterized.

Our study may provide insights into important anemia treatment patterns among ESA hyporesponders. Not unexpectedly, ESA hyporesponders required more intensive anemia treatment relative to the overall prevalent HD population. However, iron use, which was only slightly greater among the ESA hyporesponders, may represent relative underuse given that nearly 1 in 3 hyporesponders had transferrin saturations <20% at baseline. Whether this is the case, particularly when considering the baseline hemoglobin and transferrin saturation levels data of the ESA hyporesponder population, warrants further investigation. Relative underuse of IV iron may reflect the comparatively greater use of RBC transfusions in hyporesponders than in the overall prevalent HD population. Use of IV iron is highly preferable to the use of RBC transfusions for several reasons.^22^ In addition to being a valuable societal resource, blood is immunogenic, and its use sensitizes patients undergoing dialysis, making matching and favorable outcomes for kidney transplantation more challenging.^23^

Individuals with ESA hyporesponsiveness had higher unadjusted rates of all-cause mortality and of nonfatal clinical events relative to the overall prevalent HD population; our findings on mortality are concordant with previous studies.^13^^,^^24^ Cardiovascular events, when considered collectively (as thromboembolic events or MACE) or individually (heart failure, myocardial infarction, or stroke) were higher among the ESA hyporesponder subpopulation than the overall prevalent HD population, a finding previously noted.^13^ We extended these findings by examining a wide range of non-cardiovascular clinical events specifically selected to align with those reported in previous clinical trial populations receiving dialysis; unadjusted rates of key non-cardiovascular outcomes were higher in absolute terms for the ESA hyporesponder subpopulation, relative to the overall prevalent HD subpopulation. However, this study was not suited to nor designed to assess questions of causality. Future studies may wish to use different designs to evaluate potential associations between ESA hyporesponsiveness and clinical outcomes. The potential role of comorbid conditions in the clinical outcomes of patients with ESA hyporesponsiveness is also of interest for further study, noting there are generally higher frequencies of comorbid conditions in these patients than in the overall prevalent HD population.

HCRU was higher among individuals with ESA hyporesponsiveness relative to the overall prevalent HD population, consistent with a previous US study.^13^ Both the overall burden of hospitalizations and the seriousness and complexity of the hospitalizations (as indicated by use of an intensive care unit or skilled nursing facility upon discharge) were greater among ESA hyporesponders than the overall prevalent HD population. Concomitantly, physician encounters across a range of specialties, an outcome that had not been previously examined, were also more common among the ESA hyporesponders relative to the overall prevalent HD population.

Our study has several strengths. First, the use of data from the USRDS, a comprehensive registry of individuals receiving dialysis in the United States, allow our findings to be generalizable to the US HD population. Second, this study adds more contemporary evidence on patient characteristics, treatments, and clinical events in ESA hyporesponders undergoing HD in the United States. Indeed, very few studies have previously reported data on this important population.

This study also has some limitations. First, the study was not designed to determine causality and so should be considered hypothesis-generating only. Second, the definition of ESA hyporesponsiveness used in our study, which relied in part on a specific formula to establish dosage equivalency between different ESAs, was selected based on its use in recent studies, including a major clinical trial.^12^^,^^13^^,^^16^ Our results could differ if alternative definitions of hyporesponsiveness or dosage equivalency between ESA formulations were used. Third, only patients insured by Medicare fee-for-service were included in the study, meaning that patients insured by other programs were not examined. Additionally, limitations of the USRDS include some missing data on comorbidity and laboratory data at registration and possible lack of accuracy in cause-of-death reporting.^25^ Finally, there is currently no consensus on a definition for hyporesponsiveness to ESAs. The definition used in this study was selected based on its use in recent studies^12^^,^^13^; however, this varies from that of other studies and, consequently, results could differ if alternative definitions were used.

In conclusion, individuals receiving maintenance HD who are hyporesponsive to ESAs had a high prevalence of comorbid conditions and greater use of ESAs and RBC transfusions relative to the overall prevalent HD population. ESA hyporesponders had high rates not only of cardiovascular events but also of non-cardiovascular events. ESA hyporesponders had higher HCRU and encountered physicians, including primary care providers, more frequently. The findings of this study, which contribute to the greater understanding of clinical outcomes in patients who are hyporesponsive to ESAs, suggest the importance of considering their inclusion as a separate subpopulation in registry data analysis, addressing hyporesponsiveness when possible, and may help inform future resource planning.
