# Supplementary material for: Anemia Treatment and Its Clinical Implications in Patients Receiving Hemodialysis Hyporesponsive to Erythropoietin-Stimulating Agents
Source: Kidney Med. 2026 Jan 22;8(4):101269. doi: 10.1016/j.xkme.2026.101269 (PMC12966912; doi:10.1016/j.xkme.2026.101269)
Supplement: Supplementary File (PDF) — Table S1-S3. [file mmc1.pdf]

Anemia Treatment and Its Clinical Implications in Patients on Hemodialysis Hyporesponsive to Erythropoietin-Stimulating Agents

James B. Wetmore, MD, Julie Rouette, PhD, Anna Richards, PhD, Haifeng Guo, MS, Gema Requena, PhD, Suying Li, PhD, George Mu, PhD, Liyuan Ma, MS, David T. Gilbertson, PhD, Sally Wetten, MSc, and Jiannong Liu, PhD

SUPPLEMENTARY MATERIALS

Table S1. Anemia Treatment in the Follow-up Period by Patient Demographic

**Characteristic**	**ESA use,* days covered per week (95% CI)**	**IV iron use, administrations per week (95% CI)**	**RBC transfusion rate, per 100 PY (95% CI)**
**Race/Ethnicity**			
**Overall prevalent HD population**			
Non-Hispanic White	2.45 (2.44–2.45)	0.50 (0.50–0.50)	34.1 (33.7–34.4)
Non-Hispanic Black	2.63 (2.63–2.63)	0.49 (0.49–0.49)	34.4 (34.1–34.7)
Hispanic	2.83 (2.83–2.83)	0.46 (0.46–0.46)	26.4 (26.0–26.8)
Other	3.21 (3.21–3.21)	0.40 (0.40–0.40)	26.8 (26.2–27.5)
**ESA hyporesponder subpopulation**			

Non-Hispanic White	5.23 (5.22–5.24)	0.60 (0.60–0.60)	99.5 (97.6–101.6)
Non-Hispanic Black	5.23 (5.22–5.23)	0.58 (0.58–0.58)	97.1 (95.4–98.8)
Hispanic	5.24 (5.23–5.25)	0.56(0.56–0.56)	74.0 (71.5–76.5)
Other	5.31 (5.30–5.32)	0.47 (0.47–0.48)	60.0 (57.1–63.1)
**Sex**			
**Overall prevalent HD population**			
Female	2.93 (2.93–2.93)	0.49 (0.49–0.49)	35.6 (35.3–35.9)
Male	2.41 (2.40–2.41)	0.48 (0.48–0.48)	29.9 (29.7–30.2)
**ESA hyporesponder subpopulation**			
Female	5.24 (5.24–5.25)	0.55 (0.55–0.56)	87.7 (86.2–89.1)
Male	5.23 (5.22–5.24)	0.60 (0.59–0.60)	95.2 (93.6–96.8)
**Age group† (years)**			
**Overall prevalent HD population**			
18–44	2.53 (2.52–2.53)	0.50 (0.50–0.50)	34.5 (33.9–35.1)
45–64	2.56 (2.56–2.56)	0.48 (0.48–0.49)	31.3 (31.1–31.6)
65–69	2.60 (2.60–2.60)	0.47 (0.47–0.47)	32.8 (32.3–33.3)
70–74	2.65 (2.64–2.65)	0.47 (0.47–0.47)	33.5 (32.9–34.1)
75–79	2.80 (2.80–2.81)	0.49 (0.49–0.49)	34.6 (33.9–35.2)
80–84	2.89 (2.89–2.89)	0.49 (0.49–0.49)	32.2 (31.4–32.9)
≥85	3.03 (3.03–3.04)	0.47 (0.47–0.47)	29.0 (28.2–29.9)
**ESA hyporesponder subpopulation**			

18–44	5.12 (5.11–5.13)	0.54 (0.54–0.55)	100.0 (97.0–103.0)
45–64	5.23 (5.22–5.23)	0.57 (0.57–0.57)	87.4 (85.7–89.0)
65–69	5.25 (5.24–5.26)	0.58 (0.58–0.59)	93.3 (90.4–96.3)
70–74	5.27 (5.25–5.28)	0.59 (0.58–0.59)	93.4 (90.0–96.9)
75–79	5.31 (5.30–5.32)	0.60 (0.60–0.61)	98.3 (94.5–102.2)
80–84	5.35 (5.33–5.36)	0.58 (0.58–0.59)	92.4 (88.0–97.1)
≥85	5.34 (5.32–5.36)	0.56 (0.55–0.57)	68.0 (63.5–72.8)

*ESAs: Days covered per week by ESA (three epoetin alfa administration covering 1 week);

†The age groups were 18-44, 45-64, 65-69, 70-74, 75-79, 80-84, and ≥85 years. The range of values shown denotes the minimum and maximum values.

Abbreviations: CI, confidence interval; ESA, erythropoietin stimulating agents; HD, hemodialysis; IV, intravenous; PY, patient-years; RBC, red blood cell.

Table S2. Rate of Cardiovascular and Non-cardiovascular Events Per 100 PY (95% CI)

	**Overall prevalent HD population N=209,408**	**ESA hyporesponder subpopulation n=20,223**
Death (all-cause mortality)	16.9 (16.8–17.1)	26.2 (25.6–26.8)
**Cardiovascular events***		
Thromboembolic event[†]	71.0 (70.6–71.4)	80.7 (79.3–82.2)
MACE + heart failure[‡]	27.1 (26.9–27.3)	42.1 (41.3–42.9)
MACE[§]	20.3 (20.2–20.5)	30.2 (29.6–30.9)
Heart failure	9.2 (9.1–9.3)	15.0 (14.5–15.5)
Myocardial infarction	3.4 (3.4–3.5)	4.2 (4.0–4.4)
Stroke	1.3 (1.3–1.4)	1.7 (1.5–1.8)
**Non-cardiovascular events***		
Esophageal and gastric erosions	40.2 (40.0–40.5)	61.8 (60.6–63.0)
Serious infections	24.9 (24.7–25.1)	38.1 (37.3–38.9)
Cancer	5.3 (5.2–5.4)	6.6 (6.3–6.9)
Seizures	5.0 (4.9–5.0)	9.4 (9.0–9.7)

Hepatic injury	0.3 (0.3–0.3)	0.8 (0.7–0.9)
Ocular events	28.5 (28.3–28.7)	28.0 (27.3–28.7)
Retinal hemorrhage	4.6 (4.5–4.6)	4.7 (4.5–5.0)

*First event rates were calculated as the number of first events per 100 PY; †Thromboembolic event was defined as vascular access thrombosis, deep vein thrombosis, and pulmonary embolism; ‡MACE + heart failure was defined as all-cause mortality, myocardial infarction, stroke, or heart failure. §MACE was defined as all-cause mortality, myocardial infarction, and stroke.

Abbreviations: CI, confidence interval; ESA, erythropoietin stimulating agents; HD, hemodialysis; MACE, major adverse cardiovascular event; PY, patient years.

Table S3. Rates of HCRU in the ESA Hyporesponder Subpopulation Per PY*

**Healthcare services**		**Overall prevalent HD population N=209,408**	**ESA hyporesponder subpopulation n=20,223**
**Service setting**	Hospitalization	1.56	2.45
	Hospitalization with intensive care unit stay	0.37	0.61
	Hospitalization with intermediate care unit	0.54	0.86
	Hospital observation stay	0.30	0.45
	Skilled nursing facility stay	0.59	0.84
	Emergency department encounter	1.39	1.85
	Outpatient encounter	6.44	7.85
**Physician specialty**[†]	Nephrologist	15.55	18.77
	Cardiologist	4.10	5.51
	Endocrinologist	0.28	0.34

Primary care providers	10.55	14.40

*Measured as the total number of services provided/total follow-up (rate per PY); †Patients could have visited more than one physician specialty.

Abbreviations: ESA, erythropoietin stimulating agent; HCRU, health care resource utilization; HD, hemodialysis; PY, patient years.
